# Two Years of Gynecomastia Caused by Leydig Cell Tumor

**DOI:** 10.1155/2018/7202560

**Published:** 2018-07-19

**Authors:** Philip Zeuschner, Christian Veith, Johannes Linxweiler, Michael Stöckle, Julia Heinzelbecker

**Affiliations:** ^1^Department of Urology and Pediatric Urology, Saarland University, Homburg/Saar, Germany; ^2^Department of Pathology, Saarland University, Homburg/Saar, Germany

## Abstract

Gynecomastia is a common incidental finding in males that can be caused by various benign or malignant diseases. In rare cases, it results from Leydig cell tumors, a rare entity accounting for 3% of all testicular neoplasms. Some of them are hormonally active but seldom cause symptomatic endocrine disturbance. Here we report on a 32-year-old male presenting with gynecomastia which he had already been suffering from for two years. Although he had been seen by three other specialists, including a urologist, none of them found the small mass in the upper pole of his right testis. We decided to perform testis-sparing surgery which confirmed the diagnosis of a hormonally active Leydig cell tumor. During follow-up, hormonal status normalized, and gynecomastia began to resolve.

## 1. Introduction

For many urologists, gynecomastia is used as a general term for breast tissue enlargement, most often occurring as a side effect of androgen deprivation therapy for prostate cancer with a cumulative prevalence of up to 70% [1]. Nonetheless, gynecomastia can be found in approximately 50% of all males, irrespective of age and underlying clinical conditions [2]. On palpation,* true* gynecomastia, defined as proliferation of the mammary gland ductal epithelium, presents as firm and rubbery mound of tissue concentrically expanding from the nipple-areolar complex. It has to be distinguished from pseudogynecomastia, the sole accumulation of fat without any glandular proliferation [1]. Irrespective of its causes, breast tissue enlargement can be painful and bothersome, associated with psychosocial consequences, such as depression, body dissatisfaction, and reduced self-esteem [3].

Gynecomastia results from an impaired balance between free estrogen and androgen action in breast tissue with relatively elevated estrogen due to hormonal alterations on multiple levels:* (1) decreased androgen production* resulting from primary or secondary hypogonadism,* (2) increased estrogen production* from intra- or extragonadal germ cell, gastric or renal cell, or adrenal or large-cell lung tumors, or* (3) increase of aromatase* activity leading to higher conversion from testosterone to estradiol due to thyrotoxicosis, Klinefelter syndrome, aging, or increased body fat [4]. Chronic kidney and liver disease or drug or alcohol intake can be potential other causes [5]. Differential diagnosis includes male breast cancer, a rare, but life-threatening disease. Other benign conditions leading to breast tissue enlargement are lipomas, cysts, lymphoplasmocytic inflammation, or hematomas [5].

Primary tumors of the testes, such as Leydig cell tumors, can also serve as uncommon reason for gynecomastia. Testicular tumors have a low incidence (3-10/100.000 men per year in Western societies) and Leydig cell tumors (LCT) or Leydigiomas as the most common type of sex cord-stromal tumors account for approximately 3% of them [6]. LCTs are most common in adults in their third to sixth decade. Most Leydigiomas are located in the testis, but extratesticular locations such as spermatic cord, epididymis, or pelvis have been reported, too [6].

Since its first report in 1895, about 250 cases of Leydig cell tumors have been published worldwide [7]. Due to its low prevalence, the etiology of LCTs is not well understood. In contrast to most other testicular tumors, cryptorchidism does not increase the risk for LCT [8]. Structural changes of the luteinizing hormone receptors or distinct somatic or inherited mutations have been linked with its tumorigenesis [9].

LCTs may be hormonally inactive or secrete a variety of hormones including testosterone, estrogen, or its derivates. Nonetheless, endocrine disturbance only causes symptoms in 20 to 40% of cases [8]. Patients may present with a (painful) testicular mass irrespective of age. Children may suffer from precocious pseudopuberty including uni- or bilateral asymmetric gynecomastia and adults from erectile dysfunction, decreased libido, or infertility [7].

## 2. Case Presentation

A 32-year-old male patient presented to our department due to gynecomastia and breast pain he had been suffering from for 2 years. The patient had already been seen by physicians from three different specialties before, including a urologist.

More than one year earlier, a gynecologist had performed breast ultrasound and described bilateral, mainly retromammillar gynecomastia. He classified his findings as grade 3 according to BIRADS (breast imaging reporting and data system) with a risk of malignancy not higher than 2% and suggested performing a biopsy and urological evaluation.

The patient went to see an endocrinologist next who diagnosed hypogonadotropic hypogonadism with elevated estradiol and prolactin levels (Table 1). On Magnetic Resonance Imaging (MRI) of the neurocranium, no abnormalities were found. The endocrinologist suggested controlling the hormone status and pointed out possible provocation tests to further specify the findings.

Lastly, the patient had been seen by a urologist in private practice. Physical exam, ultrasound of the abdomen, and MRI of the upper abdomen did not lead to diagnosis.

**Figure 1:**
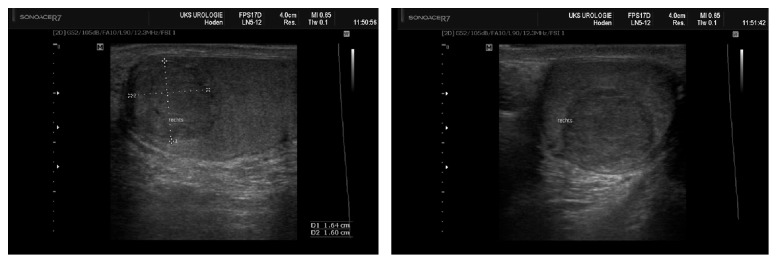
Hypoechogenic tumor in the upper pole of the right testis, measuring 1.6x1.6 cm, shown in sagittal (A) and transversal (B) plane.

In our department, the patient reported a maldescensus testis in childhood which had resolved spontaneously. He had not undergone prior surgery and did not report any regular drug intake. Physical examination did not reveal abnormalities apart from bilateral gynecomastia. On ultrasound, a 1.6x1.6 cm hypoechogenic mass within the right apical testis without hypervascularisation was detected (Figure 1).

**Figure 2:**
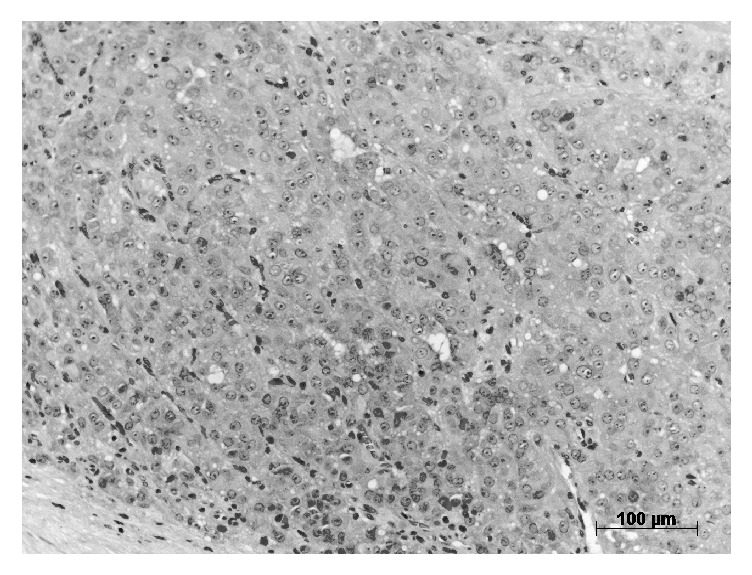
Small round cells in nests of clusters and intervening capillaries, typical for Leydig cell tumors (Hematoxylin-Eosin staining, 20x).

Considering hormonal alterations, gynecomastia, and normal testicular tumor markers, we decided to perform testis-sparing surgery with frozen section using an inguinal approach. In the operating room, the tumor appeared to be capped and rather not malignant on frozen section and could be excised in sano. Final histology confirmed a Leydig cell tumor without histological signs of malignancy (Figure 2). As chest and abdominal computed tomography did not show abnormalities, it could be classified as low risk.

On the first follow-up one month after surgery, the patient was in good general condition, yet gynecomastia had not regressed. Sexual hormones were within normal range. Half a year later, the patient had undergone a lifestyle change and lost 12 kg. Gynecomastia was still palpable but had significantly decreased. We recommended biannual follow-up for the first two years, and then yearly check-ups, including control of hormone levels, physical examination, and imaging of the chest and abdomen every 2 years.

## 3. Discussion

Leydig cell tumors (LCTs) are comparably rare amongst urological tumor entities accounting for approximately 3% of testicular neoplasms [6]. They can present with typical symptoms of testicular masses and endocrine alterations or stay completely asymptomatic [10]. In our case, symptomatic gynecomastia was the only prominent symptom—apart from a barely palpable testicular mass. For LCTs, gynecomastia is an infrequent symptom; its prevalence in larger cohorts is not higher than 10% [8].

In the presented case, gynecomastia was caused by persistently elevated estradiol, produced by Leydig tumor cells. High levels of serum-estradiol suppressed secretion of luteinizing and follicle-stimulating hormone due to negative feedback and caused hypogonadotropic hypogonadism which resolved after surgery (Table 1). However, gynecomastia did not completely regress within 6 months after surgery. This often takes more than 1 year, and sometimes surgical treatment is needed [7].

The reasons why detection of LCT in this case took almost 2 years are diverse: surely, the testicular mass was small and therefore barely palpable. Although the first medical specialist involved suggested further urological evaluation, the patient decided to see an endocrinologist next. Nonetheless, the urologist as the third specialist involved did not perform testicular ultrasound to rule out a tumor but considered adrenal malignancy instead. This underscores the importance of regular testicular ultrasound in patients in which testicular tumors are suspected or shall be excluded. The incidence of Leydig cell tumors has increased over the last decades due to better imaging techniques [8]. Other data indicate that testicular cancer incidence is increasing in general [11]. However, these data mainly refer to observations in germ cell tumors.

With the potential diagnosis of LCT in mind, we decided to perform an inguinal approach. Testis-sparing surgery is the recommended therapy for sex cord-stromal tumors of the testis; however an inguinal approach should be used as the diagnosis of testicular germ cell tumor cannot be ruled out preoperatively [12].

Most LCTs are benign. Nevertheless, about 10% of adult patients develop metastatic disease [13]. Large size (> 5 cm), old age, mitotic activity (> 3 per 10 high-power fields), vascular invasion, cytologic atypia, necrosis, infiltrating edges, extratesticular extension, and aneuploidy have been identified as putative signs of malignancy [14, 15]. The prognosis of metastatic LCTs is very poor and only retroperitoneal lymph node dissection (RPLND) has been shown to improve survival [16]. In the presented case, neither pathology nor computed tomography scans revealed signs of malignancy. For this reason, we advised the patient a biannual follow-up within the first two years after surgery, and then yearly follow-up with physical examination, hormonal status, and scrotal and abdominal ultrasound, as well as chest radiography and abdominal computed tomography every two years. Current guidelines do not make clear recommendations on follow-up of patients with benign LCTs. In high-risk patients with more than two risk factors, physical examination, hormonal status, scrotal and abdominal ultrasound, chest radiography, and CT scans shall be performed every three to six months [12, 17].

In this case report, the diagnosis of a testicular Leydig cell tumor took two years pointing out that knowledge of hormonally active testicular tumors is still low. In case of a male patient presenting with gynecomastia, every urologist should keep hormonally active testicular Leydig cell tumors in mind. Differential diagnoses are benign breast conditions, breast cancer, or endocrinopathies. In addition, every medical doctor should always try to perform thorough medical workup of his patients in order to avoid overlooking tiny, but sometimes crucial, aspects leading to diagnosis.

## Figures and Tables

**Table 1:** Laboratory results before and after surgery. After surgery, secondary hypogonadotropic hypogonadism resolved. (mo: month/months, w: week, FSH: follicle-stimulating hormone, LH: luteinizing hormone, SHBG: sex hormone-binding globulin, DHEA-S: dehydroepiandrosterone sulfate, AFP: alpha-fetoprotein, *β*-hCG: beta-human chorionic gonadotropin, and LDH: lactate dehydrogenase).

parameter (normal value)	time *before* surgery			time *after* surgery		
	10 mo	3 mo	1 w	1 mo	4 mo	10 mo
FSH (1.3 – 19.3 mIU/ml)	0.5 ↓	0.6 ↓	-	3.0	2.8	2.3
LH (1.2 – 8.6 mIU/ml)	2.8	1.6	-	7.59	8.1	5.28
Prolactin (86 – 300 *μ*IU/ml)	306 ↑	-	-	260	268	170
Estradiol (< 47 pg/ml)	82 ↑	26.2	-	-	16.6	25.7
Testosterone (1.75 – 7.81 *μ*g/l)	1.41 ↓	1.07 ↓	-	4.08	3.65	3.71
SHBG (13.2 – 89.5 nmol/l)	21.7	17.9	-	-	-	-
DHEA-S (1200 – 5200 *μ*g/l)	2590	2680	-	-	-	-
ΑFP (< 10 ng/ml)	1.8	2.5	2.5	-	2.0	2.0
*β*-hCG (< 2.71 U/l)	< 0.5	< 0.5	< 0.1	-	< 0.1	< 0.1
LDH (0-262 U/l)	-	-	231	-	184	245
